# Advances in Remediation of Environmental Pollutants in Soil–Water Systems

**DOI:** 10.3390/toxics14090838

**Published:** 2026-09-21

**Authors:** Fenghe Wang, Haotian Hao, Jiahe Miao

**Affiliations:** 1 School of Chemistry and Chemical Engineering, Nanjing University of Science and Technology, Nanjing 210094, China; 2 Key Laboratory of Drinking Water Science and Technology, Research Center for Eco-Environmental Sciences, Chinese Academy of Sciences, Beijing 100085, China; 3 Fujian Engineering and Research Center of Rural Sewage Treatment and Water Safety, Xiamen University of Technology, Xiamen 361024, China

## 1. Introduction

Soil–water systems are closely linked to ecosystem health, drinking-water security, agricultural production, food safety, and public health [1,2,3]. Industrialization, urbanization, mining, smelting, and agricultural activities can introduce metals and metalloids into soils, while wastewater discharges can transport pharmaceuticals, pesticides, industrial chemicals, and other micropollutants into surface water and groundwater [1,2,3]. The fate and biological effects of these contaminants are influenced by pH, redox conditions, mineral composition, organic matter, hydrological variability, plant growth, microbial communities, and co-occurring pollutants [3,4]. Remediation strategies should therefore be evaluated in terms of contaminant removal, underlying mechanisms, operational constraints, and practical applicability [1,4].

This Special Issue, entitled “Advances in Remediation of Environmental Pollutants in Soil–Water Systems”, examines methods and technologies for contaminant removal and risk mitigation in coupled soil–water environments. It comprises eight published papers: six research articles and two review articles. The contributions address electrokinetic remediation of heavy-metal-contaminated loess, combined organic fertilizer and phytoremediation strategies for mine tailings, resource recovery from antibiotic fermentation residues, microbial degradation of petroleum hydrocarbons and chlorinated compounds, chloride removal from strongly acidic wastewater, enhanced reductive dechlorination of carbon tetrachloride in groundwater, the migration and transformation of hexavalent chromium in soil–groundwater systems, and integrated technologies for arsenic remediation. These studies examine pollutant transport and transformation, remediation mechanisms, ecological restoration, and the development of treatment technologies for soil–water systems.

## 2. Overview of Published Articles

Metal and metalloid contamination remains a major concern in soil–water remediation because elements such as copper, lead, cadmium, chromium, and arsenic are persistent and potentially toxic. Their environmental behavior is strongly influenced by soil structure and geochemical conditions. Jin et al. investigated the effects of different chelating agents on the efficiency and mechanisms of electrokinetic remediation of Cu- and Pb-contaminated loess. The study addressed an environmental issue in northwestern China, where rapid industrialization and urbanization have intensified environmental pressures at loess sites. Comparing chelator performance helped clarify how chemical enhancement could improve electrokinetic treatment and affect Cu and Pb mobilization and removal within the mineral-pore system (Contribution 1).

Mine tailings are another important source of environmental risk, as their poor physicochemical properties and potential toxicity can limit plant establishment and ecological recovery. Wu et al. evaluated the effects of combined remediation using compound organic fertilizer and plants on microbial community structure in mine tailing substrates. Their findings indicated that tailing restoration should be assessed using plant growth, microbial community responses, and changes in substrate properties. The study supports the development of combined remediation strategies aimed at improving environmental safety and ecological function in tailing areas (Contribution 2).

Treatment approaches for industrial residues and wastewater must balance pollutant removal, risk reduction, and resource utilization. Ren et al. evaluated three treatments for the resource utilization of cephalosporin C fermentation residue. Antibiotic fermentation residue may contain high concentrations of residual antibiotics and has been listed as hazardous waste in China; effective treatment is therefore needed to reduce environmental risks. The comparison provided information relevant to the safe management and potential resource utilization of antibiotic-containing residues (Contribution 3).

Chloride removal from strongly acidic industrial wastewater remains challenging because chloride ions are highly stable under acidic aqueous conditions. Yu et al. evaluated chloride removal using ascorbic acid-assisted cuprous oxide and compared different synthesis routes. Ascorbic acid was reported to suppress side reactions involving cuprous ions, with improved chloride removal and lower residual copper concentrations. The study provided mechanistic insights relevant to the treatment of highly acidic, chloride-rich wastewater (Contribution 4).

Organic contamination in groundwater and soil–water systems was another focus of this Special Issue. Zhang et al. isolated a petroleum hydrocarbon-degrading strain and combined it with a second Pseudomonas strain to construct a dual-bacterial system. Although the system did not improve degradation under single-hydrocarbon conditions, it showed potential for treating mixtures of petroleum and chlorinated hydrocarbons. These findings suggested possible complementary interactions between the strains, although the underlying mechanisms remained to be established (Contribution 5).

Liu et al. examined carbon tetrachloride biodegradation in groundwater, focusing on microbial community dynamics and functional genes associated with enhanced reductive dechlorination. Because carbon tetrachloride is toxic and volatile, its presence in groundwater poses a potential threat to ecosystems and human health. The study provided insights into the microbial processes involved in reductive dechlorination and evidence to support the development of bioremediation strategies for chlorinated solvent contamination (Contribution 6).

The two review articles in this Special Issue broaden the mechanistic and technological context of soil–water remediation. Zhao et al. reviewed the major factors affecting the migration and transformation of hexavalent chromium in soil–groundwater systems, including soil minerals, soil organisms, hydrological conditions, microbial communities, pH, temperature, and oxygen availability. The review highlighted the combined influence of physical, chemical, and biological processes on chromium behavior and provided a framework for understanding chromium transport and guiding remediation in contaminated soil–groundwater environments (Contribution 7).

Rahman reviewed integrated strategies for arsenic remediation from wastewater, encompassing microbial, bio-based, and advanced treatment technologies. Arsenic contamination poses substantial risks to public health and environmental safety, particularly where groundwater is widely used for drinking and agriculture. By comparing these technologies, the review provided a framework for selecting and integrating remediation strategies for arsenic-contaminated wastewater and related soil–water systems (Contribution 8).

In summary, the contributions illustrate the range of pollutants and remediation challenges in soil–water environments. They address heavy-metal remediation in loess and mine tailings, resource recovery from antibiotic fermentation residues, chloride removal from strongly acidic wastewater, biodegradation of petroleum hydrocarbons and chlorinated solvents, chromium migration and transformation, and integrated arsenic remediation. Across these systems, the findings underscored the importance of considering pollutant speciation, microbial activity, material interactions, transport behavior, and process-specific limitations when selecting or designing remediation strategies for complex environmental conditions.

## 3. Future Perspectives and Concluding Remarks

The contributions to this Special Issue reflect progress in addressing diverse contaminants through a range of remediation strategies. More broadly, soil–water remediation research is moving from the assessment of individual technologies toward approaches that combine mechanistic understanding, process integration, and risk evaluation [1,4,5]. Several gaps remain. Many methods are evaluated under simplified laboratory conditions, whereas contaminated sites may contain mixed pollutants, heterogeneous soils, fluctuating hydrological conditions, and complex microbial communities [3,6]. More pilot- and field-scale studies, together with long-term evaluations of treatment stability and performance, are needed to determine whether laboratory-scale improvements translate into reliable remediation practice [6].

Future research should further examine the relationship between contaminant removal and risk reduction. Reductions in total concentration alone may not reflect changes in toxicity, mobility, bioavailability, or exposure potential [3,4]. For metals and metalloids, total content should therefore be considered alongside speciation, mobility or leachability, bioavailability, and plant uptake [3,4]. For organic contaminants, assessment should include degradation pathways, transformation products, and residual toxicity after treatment [7]. Likewise, processes for treating industrial residues and strongly acidic wastewater should be evaluated beyond removal efficiency. Relevant considerations include secondary environmental impacts, resource use, opportunities for safe resource recovery, and the stability of treated residues or precipitated products during disposal or reuse [5].

Another priority is to develop integrated remediation systems that combine the strengths of complementary approaches. Chemical, biological, physical, electrochemical, plant-based, and material-assisted technologies have different advantages and practical constraints [1,5]. Combining these approaches may improve treatment performance and ecological compatibility, but the benefits depend on mechanistic understanding and effective process integration [1,5]. Advanced materials, microbial and plant–microbe systems, electrokinetic treatment, and bio-based technologies should be evaluated in terms of selectivity, durability, cost, environmental safety, and scalability, as well as pollutant removal [1,3,5]. Life-cycle and techno-economic assessments can help identify trade-offs among remediation benefits, resource demand, and secondary environmental burdens [5].

Data-driven tools and mechanistic modeling can also support remediation research. Reactive transport and geochemical models link fluid flow, contaminant movement, and biogeochemical reactions within a common framework [8]. Microbial functional analyses and omics approaches provide complementary information about the microorganisms, genes, and transformation pathways involved in contaminant removal [9]. Machine learning may support contamination mapping, treatment design, parameter optimization, monitoring, and site-specific decisions [10]. The usefulness of these approaches depends on representative, well-integrated laboratory and field datasets. For practical applications, model outputs should provide information that environmental managers, engineers, and policymakers can use in decision-making [10].

Collectively, the studies in this Special Issue examine the performance of remediation technologies in soil–water systems, the mechanisms underlying their effectiveness, and the limitations affecting their application. Sustainable remediation should be assessed not only by contaminant removal but also by risk reduction, ecological recovery, public health protection, and feasibility under practical management conditions [3,4,5]. We hope that these contributions support further research on integrated, risk-based remediation technologies suitable for field application.

## Data Availability

No new data were created or analyzed in this study. Data sharing is not applicable to this article.
